# A Preprocess Method of External Disturbance Suppression for Carotid Wall Motion Estimation Using Local Phase and Orientation of B-Mode Ultrasound Sequences

**DOI:** 10.1155/2019/6547982

**Published:** 2019-11-21

**Authors:** Qinghui Zhang, Junqiu Li, Zhenping Qiang, Libo He

**Affiliations:** ^1^College of Big Data and Intelligent Engineering, Southwest Forestry University, Kunming 650224, China; ^2^Information Security College, Yunnan Police College, Kunming 650223, China

## Abstract

Estimating the motions of the common carotid artery wall plays a very important role in early diagnosis of the carotid atherosclerotic disease. However, the disturbances caused by either the instability of the probe operator or the breathing of subjects degrade the estimation accuracy of arterial wall motion when performing speckle tracking on the B-mode ultrasound images. In this paper, we propose a global registration method to suppress external disturbances before motion estimation. The local vector images, transformed from B-mode images, were used for registration. To take advantage of both the structural information from the local phase and the geometric information from the local orientation, we proposed a confidence coefficient to combine them two. Furthermore, we altered the speckle reducing anisotropic diffusion filter to improve the performance of disturbance suppression. We compared this method with schemes of extracting wall displacement directly from B-mode or phase images. The results show that this scheme can effectively suppress the disturbances and significantly improve the estimation accuracy.

## 1. Introduction

Nowadays, cardiovascular disease (CVD) has become one of the deadliest diseases in the world. In 2016, 17.9 million deaths were caused by CVD, accounting for 35% of the 57 million in total [1]. It is generally accepted that the early state of a carotid artery is a useful predictor of the risk of both ischemic stroke and coronary heart disease in the asymptomatic population [2, 3]. The increase of arterial wall stiffness is considered a common pathologic mechanism for many factors associated with CVD [4, 5]. Therefore, many efforts have been made to measure the stiffness of the carotid arterial wall to assess the degree of atherosclerotic disease for early diagnosis [6, 7]. In general, vascular stiffness may be expressed as some indices, such as distensibility, compliance, Peterson's elastic modulus, or Young's elastic modulus, which all can be derived from pressure and diameter measurements [8]. Therefore, the measurement of motions of an arterial wall plays a significant role in revealing the pathogenesis of the atherosclerotic carotid disease [9, 10].

The ultrasonic echography has become one of the most important noninvasive diagnostic methods for detecting and monitoring cardiovascular diseases because of technological advances in ultrasound imaging. Compared with other available imaging methods, it is a quick, radiation-free, and relatively inexpensive method of visualizing the arterial wall in vivo [11]. In the past decade, three main ultrasonic imaging methods were used for measuring displacements of an arterial wall: echo tracking [12, 13], B-mode [14, 15], and M-mode [7, 16]. Among them, the echo-tracking imaging method has a high resolution and thus has been widely adopted. However, its susceptibility to test environmental factors leads to poor reproducibility results between different measurements [16]. As for M-mode, its method of obtaining the velocity using the autocorrelation extraction envelope discards phase information which may be useful. The B-mode ultrasound image can more intuitively demonstrate the motion status of an arterial wall and offer more spatial information because of its two-dimensional display of the tissue structure. Therefore, it has attracted more and more investigations and clinical applications.

In research and clinical practice, researchers usually estimate the displacement of an arterial wall by speckle tracking generally based on a block-matching (BM) method [17–19]. This method is implemented by firstly selecting a region of interest (ROI, treated as the reference) with a specific size in the first frame of a B-mode image sequence. Then, a block showing the highest similarity to the reference block is identified from a search region around the ROI of the target B-mode ultrasound image (called the floating image), according to a prespecified measurement criterion. The coordinate difference between the two blocks represents the displacement of the wall tissue during a time step [20]. Typically, the maximization of mutual information of the intensity between the reference and a floating block is used for similarity measuring [21, 22]. Finally, the motion trajectory will be obtained after all the successive frames have been matched with the reference image.

Researchers have made many efforts to improve the accuracy of the wall motion estimation while using the BM method [23]. Some of them have investigated the influence of the shape and size of the ROI on estimation results and evaluated different sizes and shapes of the ROI [17,24–26]. The results show that the dimensions of the ROI are important factors when the block-matching method is used. A team has introduced a new speckle-tracking scheme, called multiblock matching (MBM), which uses a group of 16 blocks to estimate the global motion of the entire length of the wall [27, 28]. This method exhibits higher robustness because it is not susceptible to cumulative errors and outliers. However, this method introduces higher time and computation cost. Recently, Kalman filters have been widely applied to the BM method. They are employed to update the reference block after the block matching and improve motion detection during the block matching [29]. It has been proved that a better tracking performance can be obtained by updating either the appearance or the position of the reference block in a Kalman-based method [30, 31]. Furthermore, some researchers improve the robustness of the motion estimation of the carotid artery wall by adding a control signal into the state equation [32], introducing a state-space approach [33–35] or using an H_∞_ filter [36]. Recently, because of its importance, the estimation of the longitudinal motion of the arterial wall has attracted several researchers' interest [37]. Based on the Kalman filter method, they obtained the wall motion estimation in this direction with better accuracy [37–39].

Although those methods improve the accuracy of the block matching to some extent, external disturbances introduced during a clinical measurement have not been considered. During a clinical measurement, the obtained ultrasonic images are inevitably affected by external disturbances such as jitters of a probe operation introduced by an operator and a body position change or a respiratory motion of a test subject, which leads to significant variations between successive images. Thus, the motion extracted based on the methods mentioned above is a superimposed signal of the wall motion and the external disturbances. Especially, for those Kalman-based methods, the variations will result in divergence in a motion estimation because of successive error accumulation.

In this study, we focused on how to suppress external disturbances introduced in clinical measurements. Based on our previous work, we proposed an image registration method to eliminate external disturbances before performing speckle tracking [40]. This method treats those disturbances as common-mode disturbances and treats the wall motion as differential-mode signals. And the motion of tissue in regions far away from an arterial wall is hardly influenced by the wall motion, but only by those common-mode disturbances. Thus, we extracted the disturbances from images by performing registration on these regions. We used a local information image for registration instead of ultrasound images because their natural properties (speckle noise, high attenuation, and low contrast) will degrade the performance of the registration. To take advantage of both the structural information from the local phase and the geometric information from the local orientation [41], we proposed a confidence coefficient to combine the local orientation and local phase. Furthermore, we altered the SRAD filter before registration to improve the longitudinal disturbance suppression performance. Finally, we utilized the BM method to estimate the arterial wall motion after performing alignment on ultrasound images based on obtained disturbances.

## 2. Methodology

**Figure 1:**
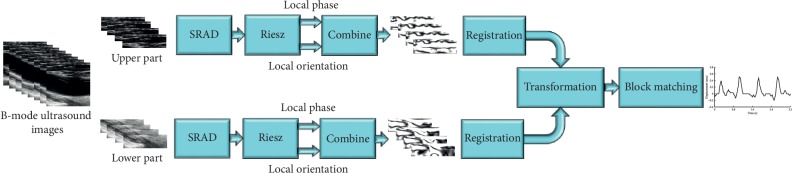
General workflow of the study.

The proposed algorithm was developed using the MATLAB software on a workstation with two Intel Xeon 8-core 64-bit processors (1.8 GHz) and 128 GB RAM. The general workflow of our study is shown in Figure 1. Firstly, two subimage sequences are cut out from original successive frames since the registration focuses on regions in an image far away from the arterial wall. Each subimage only covers the upper or lower part of the tissue in a frame, respectively, which contains abundant structural and geometric information. Because of the inherent speckles, ultrasound images will reveal large structural information better after being smoothed [42]. Besides, registration on images at a coarser level with a large structure will lead to better results. Hence, secondly, an altered speckle-reducing anisotropic diffusion (SRAD) filter is adopted to suppress the speckle noise while preserving textural information. Thirdly, the Riesz transform is used to obtain local phase and orientation information. In the next step, the registration is performed on the combined phase image sequences to obtain disturbance values. Then, all images are aligned by spatial transformation based on the average value of obtained upper and lower disturbances. Thus, the common-mode disturbances are suppressed. Finally, the motion of the arterial wall is estimated by implementing the block-matching method on the aligned successive frames. In the following sections, specific descriptions of the procedure are presented.

### 2.1. Speckle-Reducing Anisotropic Diffusion (SRAD) Filter

Because the gradient of intensity is highly sensitive to noise [43], and the texture appearance of the observed speckle does not correspond to the underlying tissue structure [44], the ultrasound image should be prefiltered to reduce the speckle noise before registration. The filter based on the SRAD method was adopted in this work, which not only suppresses speckle noise but also preserves the texture details and enhances the edges of the image [45]. This approach of SRAD is based on a partial differential equation (PDE), which is shown as follows:

(1) $$\begin{array}{c} \frac{\partial I\left(x,y;\;t\right)}{\partial t}=\text{div}\left[c\left(q\right)\nabla I\left(x,y;\;t\right)\right],\\ I\left(x,y;\;0\right)=I_{0}\left(x,y\right),\;\left({\left.\frac{\partial I\left(x,y;\;t\right)}{\vec{\partial n}}\right|}_{\partial \Omega }=0\right), \end{array}$$

where *I*_0_(*x*, *y*) denotes the given image over image support *Ω*; ∂*Ω* is the border of *Ω*; $\vec{n}$ indicates the outer normal to ∂*Ω*; div and ∇ are the divergence and the gradient operator, respectively; and *c*(*q*) is the diffusion coefficient which can be calculated according to the following equation:

(2) $$\begin{array}{c} c\left(q\right)=\frac{1}{1+\left(\left[q^{2}\left(x,y;t\right)-q_{0}^{2}\left(t\right)\right]/\left[q_{0}^{2}\left(t\right)\left(1+q_{0}^{2}\left(t\right)\right)\right]\right)}, \end{array}$$

where *q*(*x*, *y*; *t*) indicates the instantaneous coefficient of variation, treated as an edge detector in an image. It will produce high values at an edge or on high-contrast features while generate low values in homogeneous regions. It can be computed using the following equation:

(3) $$\begin{array}{c} q\left(x,y;t\right)=\sqrt{\frac{\left(1/2\right){\left(\left|\nabla I\right|/I\right)}^{2}-\left(1/4^{2}\right){\left(\nabla^{2}I/I\right)}^{2}}{{\left(1+\left(1/4\right)\left(\nabla^{2}I/I\right)\right)}^{2}}.} \end{array}$$

*q*_0_(*t*) in equation (2) denotes the speckle scale function which controls the amount of smoothing applied to the image by the SRAD and can be calculated using the following equation:

(4) $$\begin{array}{c} q_{0}\left(t\right)=\frac{\sqrt{\mathrm{var}\left[z\left(t\right)\right]}}{\bar{z\left(t\right)}}, \end{array}$$

where var[*z*(*t*)] and $\bar{z\left(t\right)}$ are the variance and mean of the intensity over a homogeneous area at *t*, respectively.

This PDE can be numerically solved using an iterative Jacobi method. The derivative and the Laplacian item in the PDE can be approximately calculated with four nearest neighboring pixels, as shown in the following equations:

(5) $$\begin{array}{c} \nabla_{R}I_{i,j}^{n}=\left[\frac{I_{i+1,j}^{n}-I_{i,j}^{n}}{h},\frac{I_{i,j+1}^{n}-I_{i,j}^{n}}{h}\right],\\ \nabla_{L}I_{i,j}^{n}=\left[\frac{I_{i,j}^{n}-I_{i-1,j}^{n}}{h},\frac{I_{i,j}^{n}-I_{i,j-1}^{n}}{h}\right],\\ \nabla^{2}I_{i,j}^{n}=\frac{I_{i+1,j}^{n}+I_{i-1,j}^{n}+I_{i,j+1}^{n}+I_{i,j-1}^{n}-4I_{i,j}^{n}}{h^{2}}. \end{array}$$

The longitudinal registration is more difficult because of the higher homogeneity of image intensities in this direction [27, 32]. To preserve more longitudinal details during the SRAD process, Aja-Fernández and Alberola-López introduced a detail-preserving anisotropic diffusion (DPAD) method [46]. In this method, a larger neighborhood was adopted to preserve more details, although it does not match equation (3). Inspired by this method, we made an alteration to equation (5) to increase the longitudinal variation, although it will not match equation (3) either. As shown in equation (6), we increased the weight of the longitudinal derivative item to 2, and the Laplacian item will be changed too:

(6) $$\begin{array}{c} \nabla_{R}I_{i,j}^{n}=\left[\frac{2\left(I_{i+1,j}^{n}-I_{i,j}^{n}\right)}{h},\frac{I_{i,j+1}^{n}-I_{i,j}^{n}}{h}\right],\\ \nabla_{L}I_{i,j}^{n}=\left[\frac{2\left(I_{i,j}^{n}-I_{i-1,j}^{n}\right)}{h},\frac{I_{i,j}^{n}-I_{i,j-1}^{n}}{h}\right],\\ \nabla^{2}I_{i,j}^{n}=\frac{2I_{i+1,j}^{n}+2I_{i-1,j}^{n}+I_{i,j+1}^{n}+I_{i,j-1}^{n}-6I_{i,j}^{n}}{h^{2}}. \end{array}$$

In this work, the homogeneous area was almost selected automatically, and the size of it is 20 × 20 pixels. To better preserve the details of the edge, according to our tests, the time step was set to 0.1, and the number of iterations was 100.

### 2.2. Local Phase and Local Orientation

Because an ultrasound image has the properties of low signal-to-noise (SNR) ratio, low image contrast, and high artifact, its registration is challenging. However, the local phase and local orientation are very suitable for the registration of ultrasound images owing to their invariance to image brightness, contrast, and noise [43]. Furthermore, they can show more local structural and geometric information than the gradient and intensity information does. Thus, the registration based on them has better stability and robustness [47, 48]. In our study, we utilized the Riesz transform to obtain local phase and orientation information of ultrasound images. To a 1D signal *f*(*x*), its complex analytic signal, defined as equation (7), is employed to extract the local information, such as amplitude and phase:

(7) $$\begin{array}{c} f_{a}\left(x\right)=f\left(x\right)+jHf\left(x\right)=A\left(x\right)e^{j\phi \left(x\right)}, \end{array}$$

where *Hf*(*x*) is the Hilbert transform of *f*(*x*), *A*(*x*) is the local amplitude, and *φ*(*x*) is the local phase. An analytic signal is a useful tool for AM/FM analysis; with it, one can obtain the time-varying amplitude and phase of a 1D signal. The monogenic signal is a 2D generalization of the analytic signal, while the Riesz transform is the 2D extension of the Hilbert transform [49, 50]. The Riesz transform consists of two kernels which can be expressed as

(8) $$\begin{array}{c} h_{x}\left(x,y\right)=\frac{x}{2\pi {\left(x^{2}+y^{2}\right)}^{3/2}},\\ h_{y}\left(x,y\right)=\frac{y}{2\pi {\left(x^{2}+y^{2}\right)}^{3/2}}, \end{array}$$

and its frequency-domain representation is shown as

(9) $$\begin{array}{c} H_{u}\left(u,v\right)=-j\left(\frac{u}{{\left(u^{2}+v^{2}\right)}^{1/2}}\right),\\ H_{v}\left(u,v\right)=-j\left(\frac{v}{{\left(u^{2}+v^{2}\right)}^{1/2}}\right). \end{array}$$

**Figure 2:**
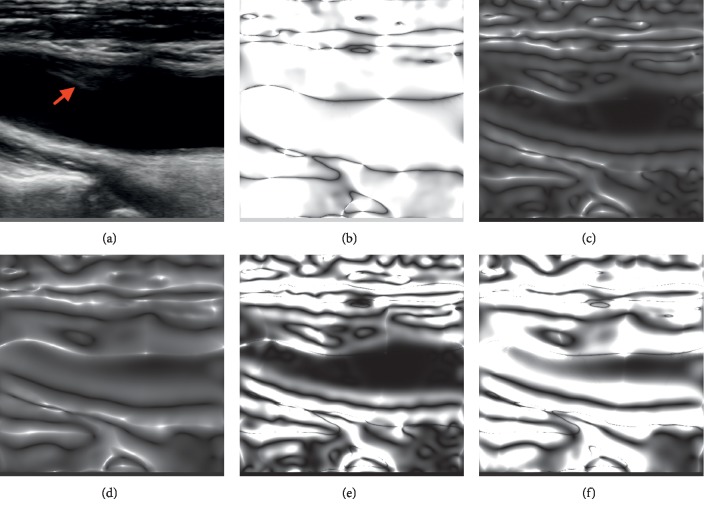
B-mode ultrasound image (a) and its local orientation images (b) and local phase images (c, d). Local orientation image with a confidence coefficient (e, f), with a smaller standard deviation ($\sigma_{1}=10\text{ and }\sigma_{2}=10\sqrt{2}$) (c, e), and with a larger standard deviation ($\sigma_{1}=20\text{ and }\sigma_{2}=20\sqrt{2}$) (d, f).

Then, the monogenic signal including three components can be described as

(10) $$\begin{array}{c} f_{M}\left(x,y\right)=\left(f,f_{R}\right)\left(x,y\right)=\left(f,\;h_{x}\left\{f\right\},\;h_{y}\left\{f\right\}\right)\left(x,y\right), \end{array}$$

where *f*_*R*_(*x*,  *y*)=(*h* *∗* *f*)(*x*, *y*) and *h*=(*h*_*x*_, *h*_*y*_). The local phase vector can be described as

(11) $$\begin{array}{c} \varphi =\arctan\left(\sqrt{h_{x}^{2}\left\{f\right\}+h_{y}^{2}\left\{f\right\}},f\right),\;\;\varphi \in \left[0,\pi \right),\\ \theta =\arctan\left(\frac{h_{y}\left\{f\right\}}{h_{x}\left\{f\right\}}\right),\;\;\theta \in \left[0,\pi \right),\\ A=\sqrt{f^{2}\left(x,y\right)+\;{\left|f_{R}\left(x,\;y\right)\right|}^{2},} \end{array}$$

where *ϕ* is the local phase, *θ* is the local orientation, and *A* is the local magnitude. Because the length of a signal must be finite in practical applications, a pair of bandpass filters is employed to convolve with the monogenic signal. In our work, the difference of Gaussians (DoG) filter was adopted, and it can be represented as

(12) $$\begin{array}{c} f\left(x,\mu ,\sigma_{1},\sigma_{2}\right)=\frac{1}{\sigma_{1}\sqrt{2\pi }}\exp\left(-\frac{{\left(x-\mu \right)}^{2}}{2\sigma_{1}^{2}}\right)-\frac{1}{\sigma_{2}\sqrt{2\pi }}\exp\left(-\frac{{\left(x-\mu \right)}^{2}}{2\sigma_{2}^{2}}\right), \end{array}$$

where *σ*_1_ and *σ*_2_ are the standard deviation of two Gaussian filters, respectively, which are related to texture details of the phase image. The coarser texture of the phase image will be obtained with a larger value, while the finer texture will be achieved with a smaller one. An appropriate standard deviation parameter will be helpful to extract the structural and geometric texture for registration, which is directly related to the quality of the results (Figures 2(c) and 2(d)).

We compared the ultrasound image with different phase images to demonstrate that both the local phase and orientation images are suitable for registration because of their invariance to image brightness and contrast. As shown in Figure 2, even in low-contrast areas of the image, the algorithm returns a reasonable value for local phase and orientation. For example, both the phase and orientation images demonstrate the stenosis part with lower echo in Figure 2(a) (marked with a red arrow). Especially, the local phase image provides more details of this part. The local orientation image retains coarser structural and geometric information ignoring the tiny details. In general, an image with a coarser structure is advantageous for registration because excessive details will increase the difficulty of registration and the time of optimization. However, artifacts may appear in a local orientation image at locations of the central line of ridges where the local phase is close to zero [48, 50]. Furthermore, there is a lack of longitudinal geometric information in local phase images, which will lead to poor longitudinal accuracy of results. Thus, a confidence coefficient based on the local phase, which can be quantified by sin^2^(*ϕ*), was introduced to local orientation in order to take advantage of both the local phase and local orientation and keep the coherence of the phase vector. It is a compromise between the two types of information and can combine the advantages of both. As shown in Figures 2(e) and 2(f), the local orientation figures with the confidence coefficient based on the local phase demonstrate that they not only provide the simple geometric information of local orientation but also preserve the structural information of the local phase, especially the longitudinal structural information.

### 2.3. Wall Movement Extraction

After suppressing the disturbance, we used a speckle-tracking method to extract the wall movement with the combined local information images taking advantage of their invariance features. A block of pixels (ROI) was selected in the first frame as a reference, and a window in floating images was searched to find the block with the highest similarity to the reference block. Some research results have demonstrated if the ROI is across the interface of the blood and an arterial wall, a more accurate wall motion can be extracted [17]. In our study, we selected the ROI in the same way and measured the similarity based on the normalized correlation coefficient (NCC), which is given as follows:

(13) $$\begin{array}{c} \text{NCC}=\frac{\sum_{m=1}^{M}\sum_{n=1}^{N}\left(R\left(m,n\right)-\bar{R}\right)\left(F\left(m,n\right)-\bar{F}\right)}{\sqrt{\sum_{m=1}^{M}\sum_{n=1}^{N}{\left(R\left(m,n\right)-\bar{R}\right)}^{2}\sum_{m=1}^{M}\sum_{n=1}^{N}{\left(F\left(m,n\right)-\bar{F}\right)}^{2}}}, \end{array}$$

where *R*(*m*, *n*) and *F*(*m*, *n*) represent the gray values of a certain point (*m*, *n*) in the first frame and the subsequent image to be matched, respectively; $\bar{R}$ and $\bar{F}$ denote the average of reference and float images; and *M* and N are the length and width of the ROI, respectively.

The length and width of the ROI are closely related to the accuracy of the speckle tracking. At least, the length of the ROI should be greater than the lateral resolution, and the width should be greater than the axial resolution [51]. To a certain degree, a larger ROI means a higher performance of motion tracking. However, when the region reaches a certain size, the performance will not be significantly improved and may even decrease. Moreover, with the increase of the ROI size, the time and computational costs will increase at a square level. Furthermore, research results indicate that ROIs with a greater dimension in the radial direction will lead to better performance because of a higher heterogeneity of pixel intensity in this direction [17]. Because the size of the ROI is not the focus of this work, we just set it to 60 × 80 pixels (approximately 3 mm × 4 mm) according to the amplitude of external disturbance.

### 2.4. Evaluation of Our Method

The assessment based on in vivo measurement is difficult because the signal components from the surrounding tissue and blood are not easily distinguishable, and the specific behaviors of a vessel wall are unknown. However, the computerized simulation is a useful validation method because of its high controllability and verisimilitude. It is commonly adopted to synthesize data sources for evaluating the performance of arterial wall motion tracking. So, apart from clinical data, in this study, we also utilized synthetic data to evaluate our method. We used preadded disturbances and predefined wall motions as a reference to evaluate the disturbance extraction based on registration and motion estimation based on aligned images.

We obtained clinical images using a high-resolution ultrasound system (SonixTOUCH; Ultrasonix Medical Corporation, Burnaby, British Columbia, Canada) with an L14-5W/60 linear array transducer. A total of ten subjects were measured to collect real image data (center frequency = 5 MHz, sample frequency = 40 MHz, number of elements = 128, and active elements = 64). The simulation data were generated using the software named “Field II,” which was created by Jensen [52]. The parameters of ultrasound simulation were the same as those of clinical experiments. Considering the importance of the simulated data to evaluation, we present the details about the simulation procedure in the following sections.

#### 2.4.1. Simulation Method

**Figure 3:**
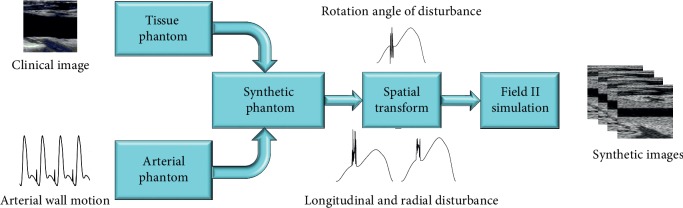
Flowchart of the synthetic process of the B-mode ultrasound image.

We used a clinical image to synthesize B-mode images to obtain more realistic images for the assessment of the registration results. The flowchart of the synthetic process is shown in Figure 3, and the specific processing steps are described as follows.

**Figure 4:**
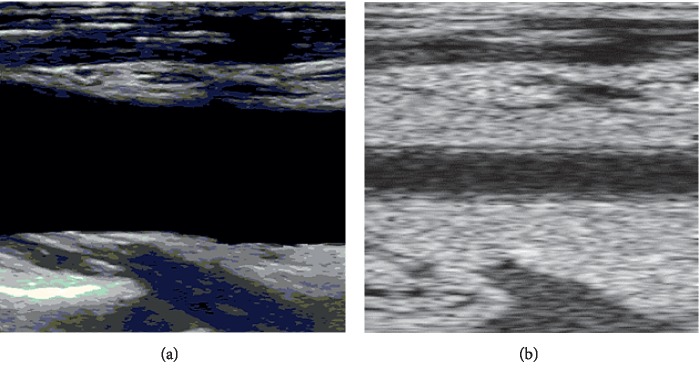
Clinical B-mode ultrasound image after removing the part of the wall (a) and the synthetic image (b).

Firstly, we removed the part near the arterial wall while keeping the part far away from the wall of a clinical vascular B-mode ultrasound image (Figure 4(a)). Then, we used the SRAD filter to preprocess the image. The phantom of the tissue part far from the arterial wall was generated, in which the amplitude of the reflecting wave of each scattering point is set up based on the gray level of the filtered image.  Figure 4(b) shows a synthesized frame whose peripheral part is very similar to the original B-mode image.

**Figure 5:**
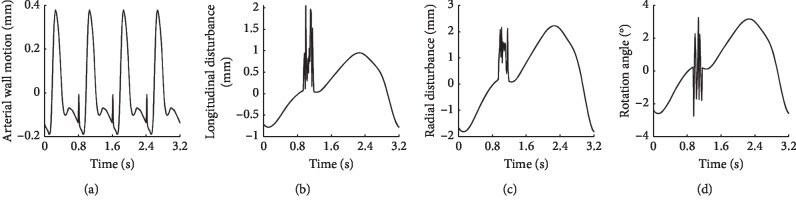
Arterial wall motion (a); external disturbances in the longitudinal direction (b) and radial direction (c); rotation angle (d).

Secondly, we established the phantom of the vascular tissue with wall pulsation. We assumed that the movement of the arterial wall, pushed by the pulsatile blood, follows the trajectory shown in Figure 5(a) which lasts a respiratory cycle (four cardiac cycles). Then, we obtained a sequence of phantoms with wall motion. The position of each scatterer in the phantoms changed along with the movement of the arterial wall, while the reflection amplitude of each scatterer was determined by its initial position. Combining the tissue phantom with the arterial phantom, we synthesized an integral phantom without external disturbance. As shown in Figure 4(b), a B-mode ultrasound image even including the intima, media, and adventitia was synthesized for better imitation of the clinical images.

Thirdly, we added external disturbance, which is caused by the breathing motion of the test subject and jitter of an operator, to the simulated 3D phantoms. As shown in Figures 5(b)–5(d), the black curves represent the longitudinal and radial displacements of the arterial wall and the angle of rotation caused by external disturbances, respectively. The horizontal axis in those figures is the time axis, and the length is 3.2 seconds (simulating a respiratory cycle). The glitch-like part of those curves is a simulation of the probe jitter caused by an operator. Then, by rotating and shifting phantoms obtained in the previous step based on the disturbances, we obtained a phantom sequence with the arterial wall motion and disturbances.

Then, we inputted this series of phantoms in Field II to generate echo signals. Finally, after performing envelope extraction, dynamic range compression, interpolation, etc. on echo signals, we synthesized a sequence of the B-mode images with disturbance.

#### 2.4.2. Comparison with Other Methods

To evaluate the disturbance suppression performance of our method (abbreviated as SWPOM), taking advantage of simulated data, we compared its results with the reference (preset disturbance). Simultaneously, we compared its results with results obtained from the registration method based on B-mode ultrasound images (BM), local phase images (PM), ultrasound and phase images after SRAD filtering (SBM and SPM) or altered SRAD filtering (SWBM and SWPM), and local orientation images after altered SRAD filtering (SWOM). Moreover, we compared the preset wall motion with the tracking results based on the aligned ultrasound image sequences.

## 3. Results

### 3.1. Simulation Results

**Figure 6:**
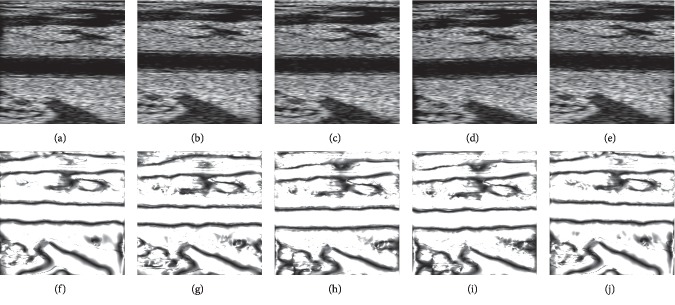
Several frames of the synthetic B-mode ultrasound sequence (a–e) and their corresponding combining phase images (f–j) with wall motion and disturbances at several time points (0 s, 0.9 s, 1.1 s, 2.2 s, and 3.2 s).

We obtained a sequence of ultrasound images (200 frames) with wall motion and external disturbance according to the procedure aforementioned. As shown in Figure 6, we selected several frames at the start, the end, and the moment that the amplitude of external disturbance is high (0 s, 0.9 s, 1.1 s, 2.2 s, and 3.2 s) to illustrate that the simulation results can reflect the influence of added disturbance. For example, by comparing the first frame with others, we could easily perceive that these images have been apparently moved and rotated. Furthermore, compared with data in Figures 6(d) and 6(i), Figures 6(a) and 6(f) show that these images had been moved downward and rotated counterclockwise at a certain angle.

#### 3.1.1. Extracted Disturbance

**Figure 7:**
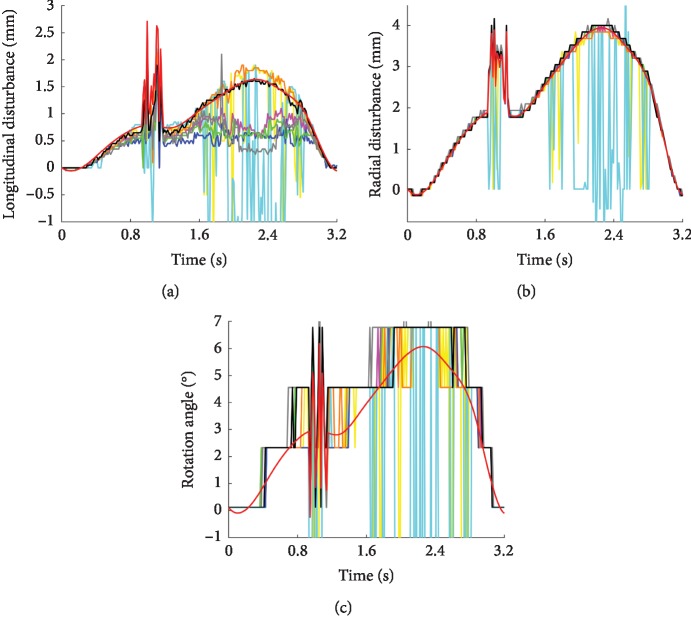
Comparison of estimated results of the disturbance over one respiratory cycle, for the longitudinal direction (a), radial direction (b), and rotation angle (c). The disturbance obtained by the BM (blue), PM (cyan), SBM (magenta), SPM (yellow), SWBM (green), SWOM (gray), SWPM (orange), and SWPOM (black) methods and the reference (red) are demonstrated.

Figure 7 qualitatively demonstrates the pointwise amplitude comparison among the reference (preset disturbance) and extracted disturbances using different image types. The obtained disturbance curves of radial direction and rotation angle generally show a better similarity to the reference than longitudinal curves do. In addition, the estimated results for both directions and rotation angle based on PM and SPM methods are poor, and there are quite a few outliers, mostly obtained at the moment that the amplitude of the external disturbance is large (for example, at the moment of about 1 second, the jitter of the probe superimposes on the signal). In general, the results based on images with SRAD filter processing are better than those without processing, while the results with altered SRAD processing are better than those with SRAD processing, especially in the longitudinal direction. Moreover, although the radial displacement and rotation angle results based on the SWPOM method are almost the same as those based on BM, SBM, SWBM, and SWPM methods, the longitudinal result, however, is better than others.  Table 1 shows the normalized root-mean-square error between the disturbance curves obtained by those methods and the reference value, which quantitatively better illustrates the performance of the SWPOM method in the longitudinal direction.

#### 3.1.2. Estimation of Arterial Wall Motion

**Figure 8:**
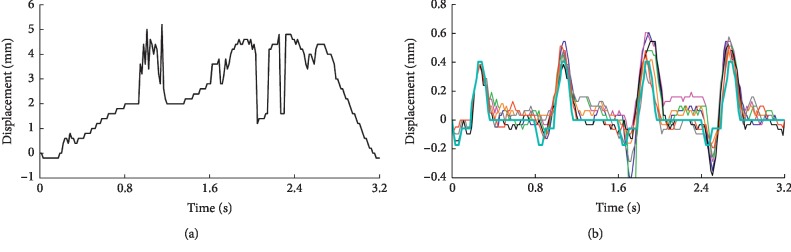
Estimated results of wall motion over one respiratory cycle without (a) and with (b) disturbance rejection processing. (b) Comparison between the wall motion estimation obtained from the image sequence without disturbance (cyan) and with disturbance but aligned. The alignments are based on the reference disturbance (red) and the estimated disturbance obtained by the BM (blue), SBM (magenta), SWBM (green) SWOM (gray), SWPM (orange), and SWPOM (black) methods.

After the B-mode ultrasound image sequence had been spatially transformed based on the obtained disturbance values, the arterial wall motion was extracted by the speckle-tracking method (Figure 8). The black curve in Figure 8(a) represents the estimated wall motion trajectory directly extracted from the B-mode image sequence with external disturbance. It clearly demonstrates the influence of external interference signals, and we cannot even tell the movement of the arterial wall itself from this curve. However, the estimations of wall motion based on the image sequences with disturbance rejection processing have similar trajectories to the curve extracted from the image sequence without external disturbance (Figure 8(b)). At the same time, the results are very similar to the reference curve. Obviously, the registration methods suppressed the disturbance, although it had not been completely removed. For example, the motion amplitude error during the last three cardiac cycles is a little high, while the error during the first cardiac cycle is low, which is obviously because of the higher disturbance amplitude during the last three cardiac cycles. Nevertheless, the errors are small because of the suppression process of disturbances. It should be noted that, in order to show a more clear comparison, the results based on PM and SPM methods are not illustrated in Figure 8(b) because of their larger errors.  Table 2 summarizes the normalized root-mean-square errors between the preset wall motion trajectory shown in Figure 5(a) and the motion estimations obtained from aligned image sequences based on different registration methods, as well as the reference disturbance. As shown, the root-mean-square error of the SWPOM method (0.5359) is the closest result to the reference one (0.5007) because of its better disturbance estimation accuracy as a whole in both directions and rotation angle.

**Figure 9:**
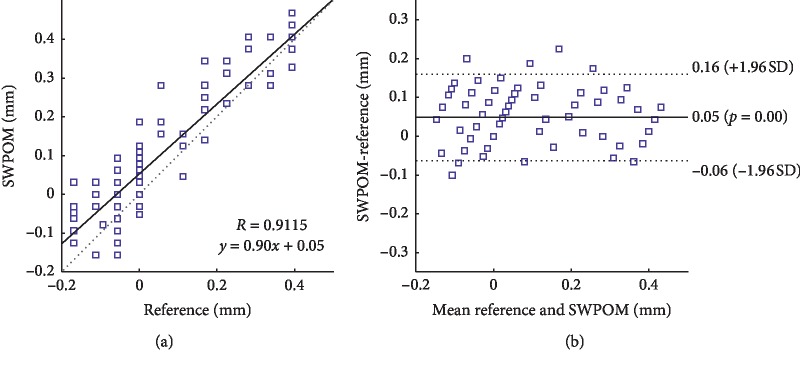
Linear regression line and correlation coefficient *R* between the reference value of the motion amplitude and the estimation performed by the SWPOM method (a); Bland–Altman plot comparing the motion amplitude estimated by our SWPOM method with the reference (REF) (b).

Furthermore, to demonstrate the disturbance suppression method has no influence on the motion tracking, the linear regression analysis and the Bland–Altman analysis were applied to measure the correlation and the agreement between the reference wall motion value and estimated results based on the disturbance suppression method. Here, the reference value indicates the wall motion estimation based on images without preset disturbance.  Figure 9(a) shows the correlation between the reference and SWPOM methods, and the correlation coefficient *R* is 0.9115, indicating a close relevance.  Table 3 lists the results of linear regression analysis over all the methods. It shows that all methods have good relevance to the reference method except for PM and SPM methods because of more outliers. As shown in Figure 9(b), a Bland–Altman plot demonstrates the agreement between the reference and SWPOM methods, where the continuous line is the mean value of difference and the upper and lower dashed lines indicate the 95% confidence interval (CI) of the mean difference (mean ± 1.96 SD). The mean difference between the reference and SWPOM methods is 0.05 mm, and the CI is 0.11 mm, which indicates that the SWPOM method has a good agreement with the reference method.

### 3.2. Results of Clinical Images

**Figure 10:**
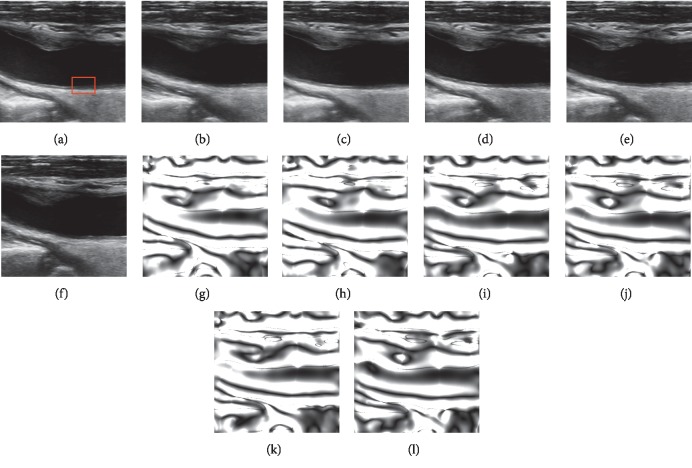
Clinical B-mode ultrasound images (a–f) and their corresponding combining phase images (g–l) at several time steps (0, 2, 4, 6, 8, and 10 seconds).

To further validate the algorithm proposed in this paper, we employed those methods to clinical B-mode ultrasound images of subjects. The duration is 10 seconds, with a total of 300 frames.  Figure 10 shows six frames of the B-mode image sequence at different time steps (0, 2, 4, 6, 8, and 10 seconds) from a patient with moderate carotid atherosclerosis, as well as their corresponding phase images. By comparing these clinical subimages, we can roughly appreciate the advantages of the combining phase image on the invariance of the brightness, contrast, and noise. For example, the stenosis part in Figures 10(b) and 10(d) is more blurry and darker, and its brightness and contrast obviously differ from those of other subimages although they have a similar shape. However, their combining phase images are almost the same because of the invariance advantage of the phase vector. Moreover, the tissues in regions far away from the arterial wall of different frames maintain a good consistency with each other. Thus, it is suitable to suppress the common mode interference by using the registration algorithm on combining phase images proposed in this paper.

#### 3.2.1. Extracted Disturbance

**Figure 11:**
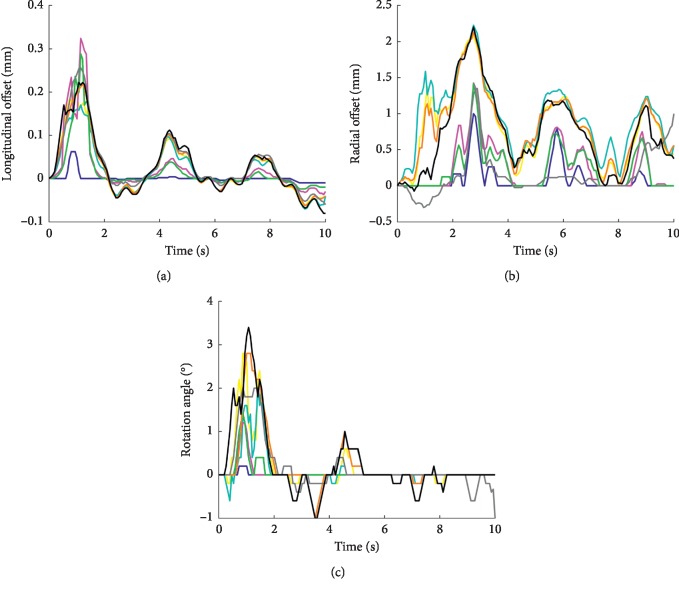
Extracted disturbances from the clinical image sequence, for the longitudinal direction (a), radial direction (b), and rotation angle (c). The disturbance obtained by the BM (blue), PM (cyan), SBM (magenta), SPM (yellow), SWBM (green) SWOM (gray), SWPM (orange), and SWPOM (black) methods is demonstrated.

Figure 11 shows the extracted disturbances for the longitudinal direction, radial direction, and rotation angle with different methods, based on the clinical ultrasound images mentioned above. All these curves reflect the trend of respiratory movement trajectories of about three cycles, which will be suppressed as common mode interferences. In addition, the amplitudes of extracted disturbances based on phase images are higher than those on B-mode ultrasound images.

#### 3.2.2. Estimated Wall Motion

**Figure 12:**
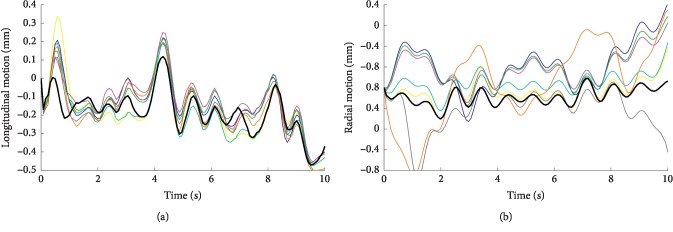
Wall motions estimated from a ten-second clinical B-mode ultrasound image sequence in longitudinal (a) and radial (b) directions after disturbance rejection processing. The disturbance rejections are based on the estimated disturbances demonstrated in Figure 12 which are obtained by the BM (blue), PM (cyan), SBM (magenta), SPM (yellow), SWBM (green), SWOM (gray), SWPM (orange), and SWPOM (black) methods.

Figure 12 demonstrates the estimation of the wall motions by the speckle-tracking method in longitudinal (Figure 12(a)) and radial (Figure 12(b)) directions after disturbance rejection processing. The red box in Figure 10(a) is the selected ROI for block matching. According to the result curve in longitudinal and radial directions, the vessel wall pulsated about 14 times. Radial results reflect better regularity of wall pulsation than longitudinal results. In addition, the results based on phase methods (PM, SPM, and SWPM) show a smoother curve trend than those based on B-mode ultrasound methods (BM, SBM, and SWBM). Specifically, the curves based on B-mode ultrasound images present a higher jitter at the time of about 1.7 s in both directions and present a little obliquely upward drift during the latter part of the time. On comparison, the result of the SWPOM method in the radial direction is almost the same as that of other methods based on the phase image. However, its result in the longitudinal direction is slightly smoother than that of others. And the estimated curves of the SWPOM method better reflect the periodicity of the arterial wall pulsation.

It is difficult to assess the performance of the suppression method used on clinical images by comparing the extracted values with the reference because we did not have an accurate reference. The arterial wall movement of subjects and the external disturbance are unknown during clinical measurement, which is different from the simulation cases. Thus, we just selected a smooth cycle from the image sequences and took it as a reference. Then, we compared it with other cycles of image sequences. This method is not for accurate assessment; however, to some extent, the results will qualitatively reflect the performance of external disturbance suppression because, in theory, the suppression method will make the results look like a smooth periodic signal.

To minimize the external disturbances during the acquisition of the reference, we asked the subjects to hold their breath and the tester to place the probe steady and gently on the neck of subjects. We collected data from ten volunteers and calculated RMSEs according to this rule.  Table 4 lists comparisons between BM and SWPOM methods for these ten subjects. The results show the RMSEs of the SWPOM method are smaller than those of the BM method in both directions, especially in the radial direction, although a different selection of the reference may lead to a different value of RMSE. Therefore, it demonstrates that the method proposed in this paper can effectively suppress the external disturbances of the clinical data.

## 4. Discussions

### 4.1. On Synthetic Ultrasound Image

In this study, the synthetic B-mode ultrasound image sequence was not obtained by simply shifting or rotating the first frame of a two-dimensional image sequence but by spatially transforming every scatterer in the phantoms generated for each time step and then calculating with the Field II software based on the phantoms. The time cost and computational overhead of the latter simulation method are much higher than those of the former one. However, because the speckle is a reflection of the differences between the image pattern and the scatterer pattern, the speckle pattern that exists in a given image of the sequence may not appear in the next frame [17]. If the ultrasound images are transformed only on a two-dimensional plane, the pattern of speckles in successive frames is almost the same except for the locations, which is impossible in a clinical situation. Thus, it may result in an unreliable assessment of the motion-tracking algorithm. Compared with the former method, the spatial transformation of each scattering point in our method was done in a three-dimensional cylindrical coordinate. The scatterers in different phantoms would keep the random distribution property because every phantom at each time step was generated independently. Thus, in spite of a higher time cost, this method will make the speckle-changing pattern in those simulated B-mode ultrasound images agree with the actual situation and be better suitable to assess the CCA wall motion-tracking algorithm.

### 4.2. On the SRAD Filter

In our work, in order to reduce the impact of the speckle's pattern changes between frames on the registration result, the SRAD was adopted. Because of its anisotropic filter property, it smoothes the speckle noise while reserving the texture and edge information, which will be helpful for registration based on gray and gradient information. We verified through experiments that the filtering performance is not sensitive to the size of the homogeneous area, time step value, and the number of iterations. However, the selection of the homogeneous area has a large impact on the image pattern. Therefore, to get a sequence of images with better consistency, we need to choose almost the same area in a sequence image. Although manual setting is a reliable method, however, it is impractical because of the large number of frames that need to be processed. So, an automatic method was implemented in this article. For the simulated data, firstly, we selected a homogeneous area in the first frame and obtained the coordinates of this region. Then, the homogeneous areas of other frames could be obtained by taking their preset disturbances as the relative offset and adding them to the coordinate of the selected region in the first frame. For clinical images, because of the unknown external disturbances, we calculated the principal axis and centroid of each frame and then roughly estimated the relative offset. This process would make sure most images maintain better consistency after SRAD filtering. A few abnormal frames were updated by reselecting the homogeneous areas manually. Because of its importance, how to automatically select an appropriate homogeneous area will be the direction of future research.

### 4.3. On This Method

In this work, we focused on the suppression of external disturbances introduced by the instability of the probe operator and the breathing of subjects, considering them as a common mode interference. Therefore, we did not compare these motion-tracking results with those of other methods mentioned in the literature but compared the extracted disturbance with the preset value. The more consistent they are, the better the performance of the method for filtering out external disturbance. Those new wall motion-tracking methods can be performed after our disturbance suppression method. To verify the suppression will not affect the motion of the arterial wall, we compared the estimation of wall motions extracted from aligned images with the reference trajectory of wall motion. Then, we used linear regression analysis and the Bland–Altman analysis to demonstrate this. The higher correlation and agreement mean that our suppression approach has little effect on wall motion. Our method is based on the assumption that the tissues far away from the vessel wall in different frames have a similar structural and geometric pattern. We found it is reasonable by reviewing many of the clinical ultrasound image sequences.

As shown in Figure 8 and Table 1, there are so many outliers in the disturbance extracted results based on the phase images (PM and SPM), which does not coincide with the advantages of the phase information. However, as shown in Figure 11, the results based on clinical images are much better. This is probably because the difference in speckle pattern detail of the successive simulated B-mode ultrasound image results in a larger difference between its phase images than that of clinical images, although they may look very similar to each other. Although the clinical phase image works well, the solution to this problem will improve the reliability and robustness of our method. This will be the direction of our future research. Moreover, although we used the altered SRAD filter, the results in the longitudinal direction are still poorer than the radial results, which is probably because of the texture characteristics of the tissues. How to improve the disturbance suppression performance in the longitudinal direction is challenging and will be the direction of our future research.

## 5. Conclusions

In this work, we proposed an approach to suppress the disturbance introduced by the instability of the probe operator and the breathing of subjects. This method is realized by registering the tissues far away from the arterial wall in ultrasound images. Firstly, the SRAD filter was utilized to remove the speckle noise while preserving the edge information. Moreover, we increased the longitudinal weight of the gradient item in the diffusion equation to promote the performance of longitudinal suppression. Then, taking advantage of the invariance to image brightness and contrast of the local phase vector, we employed a combined approach of the local phase and local orientation for registration. Clinical and simulation results show that this method can effectively suppress external disturbances without impact on the motion of the arterial wall. Combined with new motion-tracking methods, this method will further improve the estimation accuracy of motion estimation.

## Figures and Tables

**Table 1:** Normalized root-mean-square error of estimated disturbance results vs. reference disturbance.

RMSE	BM	PM	SBM	SPM	SWBM	SWOM	SWPM	SWPOM
Radial direction	0.0242	0.6662	0.0237	0.2537	0.0278	0.0268	0.0240	0.0241
Longitudinal direction	0.5460	1.1008	0.3951	0.5370	0.4924	0.9224	0.1685	0.1349
Rotation angle	0.2119	2.2425	0.3094	0.8868	0.3078	0.3682	0.2183	0.2643

**Table 2:** Normalized root-mean-square error of estimated wall motions based on the speckle-tracking method vs. reference wall motion.

	REF	BM	PM	SBM	SPM	SWBM	SWOM	SWPM	SWPOM
RMSE	0.5007	0.7360	4.7382	0.8622	1.9040	0.7668	0.6471	0.6300	0.5359

REF denotes the result obtained from aligned images based on preset disturbance.

**Table 3:** Correlation coefficient of estimated wall motions based on the speckle-tracking method vs. reference wall motion.

	REF	BM	PM	SBM	SPM	SWBM	SWOM	SWPM	SWPOM
CR	0.9121	0.8397	0.1060	0.8252	0.4718	0.7570	0.8870	0.9112	0.9115

**Table 4:** RMSEs of the SWPOM and BM methods for ten subjects.

Direction	Method	1	2	3	4	5	6	7	8	9	10
Longitudinal	SWPOM	0.0165	0.0225	0.0166	0.0161	0.0283	0.038	0.0298	0.0161	0.0307	0.026
	BM	0.0252	0.0278	0.0222	0.0303	0.0336	0.0389	0.0327	0.0303	0.426	0.0374
Radial	SWPOM	0.0125	0.0103	0.0108	0.0186	0.0166	0.0179	0.0182	0.0175	0.0186	0.0113
	BM	0.0741	0.119	0.0805	0.0959	0.1062	0.1464	0.0309	0.109	0.0832	0.0783

## Data Availability

The simulated and clinical images data used to support the findings of this study are included within the supplementary information files.

## Supplementary Materials

Supplementary Materials The first attachment file (Bmode_3layer.zip) is the compressed file of a sequence of synthesized B-mode ultrasound images. These images were generated using Field II software based on the method introduced in this paper. These images simulate a carotid wall motion with external disturbance and were used to evaluate the disturbance suppression method introduced in this paper. The second and third files (Bmode clinical(1).zip and Bmode clinical(2).zip) are compressed files of a sequence of clinical B-mode ultrasound images. These images were obtained from a B-mode video of a patient with moderate carotid atherosclerosis. We choose 300 frame images (from jm297 to jm596, the duration is 10 seconds) to assess the disturbance suppression method introduced in this paper.
